# Effect of different smoking processes on the nutritional and polycyclic aromatic hydrocarbons composition of smoked *Clarias gariepinus* and *Cyprinus carpio*

**DOI:** 10.1002/fsn3.1107

**Published:** 2019-06-11

**Authors:** Cristelle T. Tiwo, François Tchoumbougnang, Elvis Nganou, Pankaj Kumar, Binay Nayak

**Affiliations:** ^1^ Department of Biochemistry University of Dschang Dschang Cameroon; ^2^ Centrale Institute of Fishery Education Mumbai India; ^3^ Department of Biochemistry University of Douala Douala Cameroon; ^4^ Department of Pharmaceutical Chemistry, NGSM Institute of Pharmaceutical Science Nitte University Deralakatte, Mangaluru India

**Keywords:** *Clarias gariepinus*, *Cyprinus carpio*, improved smoking, nutritional quality, polycyclic aromatic hydrocarbons, traditional smoking

## Abstract

The effect of different smoking processes on the nutritional value and polycyclic aromatic hydrocarbon (PAHs) content of *Clarias gariepinus* and *Cyprinus carpio* has been assessed in this study. After smoking processes, the finish products were analyzed to determine the nutritional quality and the PAHs content. Different smoking processes significantly decreased (*p* < 0.05) lipids content of fish. The smoked fish with unfiltered *Psidium guajava* has revealed higher lipid contents of 14.17 ± 0.15% and 14.96 ± 0.05%, respectively, for SNE GSF and SE GSF. A significant reduction (*p* < 0.05) in protein content (% DM) has been observed in the two fish's species submitted to smoking processes. We found that evisceration of fish before smoking leads to increase the level of naphthalene, acenaphthene, and benzo (a) pyrene in smoked *C. gariepinus* and *C. carpio*. The use of metallic filter in the smoking of noneviscerated fish leads to the significant reduction (*p* < 0.05) of the PAHs content in smoked fishes. Higher levels of PAH such as naphthalene and acenaphthene with values of 1,451.54 ± 49.58 and 709.91 ± 8.12 ng/kg were found in smoked *C. carpio* and 1,841.1 ± 11.41 and 809.91 ± 1.10 ng/kg were found in smoked *C. gariepinus* obtained in the case of traditional smoking. The PAHs content was higher in fish smoked using traditional ovens. Therefore, the quality of smoked fish was improved using a metallic filter during different smoking processes.

## 1 INTRODUCTION

Smoking is one of the fish preservation methods that combine drying and decomposition of wood during combustion that lead to component such as phenol, formaldehyde, organic acids, and polycyclic aromatic hydrocarbons (PAHs); (Ekomy, Bruneau, Mbega, & Aregba, 2013). Smoking is mainly applied because it improves the organoleptic profile such as, color, texture, and flavor of the fish (Hitzel, Pöhlmann, Schwägele, Speer, & Jira 2012). On the other hand, it provides undesirable effects of amount which, the most important is the contamination of food by toxic and carcinogenic compounds such as PAHs, N‐nitrosamines, heterocyclic aromatic amines, and β‐carbolines (Ledesma, Rendueles, & Díaz, 2017).

Polycyclic aromatic hydrocarbons are chemical compounds that contain three or more aromatic rings. PAHs are formed by wood during combustion (Basak, Gülgün, & Telli, 2010; Wretling, Eriksson, Eskhult, & Larsson, 2010). The European Union Scientific Committee on Food (SCF) has identified 15 PAHs compounds as carcinogenic genotoxic (benz (a) anthracene, benzo (b) fluoranthene, benzo (j) fluoranthene, benzo (k) fluoranthene, benzo (a) pyrene, benzo (g,h,i) perylene, chrysene, cyclopenta (c,d) pyrene, dibenz (a,h) anthracene, dibenzo (a,e) pyrene, dibenzo (a,h) pyrene, dibenzo (a,i) pyrene, dibenzo (a,l) pyrene, indenol (1,2,3‐cd) pyrene, and 5‐methylchrysene). Benzo (α) pyrene has the highest carcinogenic value than other PAHs compounds. It contributes from 1% to 20% of total carcinogenic effects found in smoked products (European Commission, 2002; Swastawati, Winarni, Darmanto, & Nurcahya, 2007). PAHs contamination from smoked fish can be significantly decreased by improving the smoking process in others to avoid the fish to be placed directly in contact with smoke source (Visciano, Perugini, Conte, & Amorena, 2008).

The use of the traditional method with smoking kiln as an alternative smoking method has been implemented since many years in Indonesia. Indeed, the traditional smoking method can lead to PAHs contamination, so that the process needs to be controlled adequately (Leksono, Bustari, & Zulkarnaini, 2009). Kafeelah et al. (2015) in their work on the influence of fish smoking methods on the polycyclic aromatic hydrocarbon content and possible risks to humans noted that high PAH in traditional smoking methods could lead to cancer development. In the same lines, Nnaji and Ekwe (2018) found that smoking processes increased PAH levels in the catfish and tilapia muscles so that mean concentrations of benzo (a) pyrene and total PAH concentrations exceeded the limits set by the European Union of 2.0 and 10 μg/kg, respectively. Therefore, it is known that smoking and fish processing technic such as boiling, roasting, and cooking revealed that phenanthrene, naphthalene, fluorine, and acenaphthene are the most represented, while the lowest values were observed in benzo (a) pyrene, benzo (k) fluoranthene, and anthracene (Coroian et al., 2017).

Toxicological studies on individual PAHs in animals, mainly on the PAH benzo(a)pyrene, have shown various toxicological effects. One of the significant sources of PAHs in the human food chain is the smoked meat and fish. Smoke provide not only a special taste, color, and aroma to food, but also enhances preservation due to dehydration, bactericidal, and antioxidant properties of smoke (Miculis, Valdovska, Sterna, & ZutiPolycyclic, 2011). Smoking appears to be one the most used conservation technique, but there are no data regarding the reduction of the PAHs content in smoked foods. Therefore, based on the nature of PAHs, the objective of this work was to study the effect of different smoking processes using metallic filter on the nutritional and PAHs composition of smoked *Clarias gariepinus* and *Cyprinus carpio*.

## 2 MATERIAL AND METHODS

### 2.1 Smoked process

Fishes were collected at Batie of latitude 50°17′00″ North and longitude 100°17′00″ West, in the west region of Cameroon. They were smoked using three types of woods (*Mangifera indica*, *Psidium guajava*, and *Rhizophora mangle*). Many treatments were applied during the process. Fishes were smoked using an ameliorated smoking oven. Before using this smoking oven, another grid with a metallic filter of 100 µm of diameter was placed under the grid in contact with fishes and allows fishes, to be in contact only with filtered smoke. Another parameter that has been considered during this study was the evisceration of fish. This parameter was studied in order to evaluate his effect, associated with the use of metallic filter during the smoking of *C. gariepinus* and *C. carpio* using three types of woods in the ameliorated smoking oven for 7 hr. Another part of these fishes was smoked using a traditional smoking oven during 7 hr in which the temperature was up to 80°C. All these samples were analyzed and compared with control and to the smoked *C. gariepinus* and *C. carpio* purchased in the local market.

### 2.2 Proximate composition of *C. carpio* and *C. gariepinus*

The moisture, ash, lipid, and nitrogen content of crude and smoked fish were determined according to the methods described by AOAC (2016). The protein content of fish was determined by estimating its total nitrogen content using the Kjeldahl method. Nitrogen obtained was multiplied by a factor of 6.25 for the determination of the total protein content.

### 2.3 Extraction and identification of PAHs

#### 2.3.1 Extraction

Extraction and identification of PAHs has been determined according to the method described by Takasuki et al. (1985). Ten gram of dried fish sample was introduced into a bottom flask containing 200 ml of ethanol, 35 ml of a 50% aqueous solution of KOH, and 2 g of Na_2_S_9_H_2_O (sodium sulfide monohydrate). The mixture was refluxed for 2 hr on the hot plate. At the end of this step, the solution was cooled and maintained at 40°C of temperature. One hundred milliliter of n‐hexane was added in the mixture with slight swirling to allow homogenization. The mixture was transferred into a 500‐ml separating funnel containing 100 ml of distilled water. The flask was rinsed with 50 ml of n‐hexane. The mixture has been stirred vigorously. The mixture could stay in the separating funnel for the separation in two phases. The solution was extracted with 150 and 100 ml of n‐hexane, respectively. The top layer, especially hexane, was collected and filtered through anhydrous sodium sulfate. The filtrate was concentrated using a rotary evaporator to a final volume of 3–5 ml.

#### 2.3.2 Filtration of PAHs

The chromatographic column (20 mm ID) was used in this filtration step. Eight gram of silica gel was introduced into the column. After that, 3 g of anhydrous sodium sulfate was added to cover the silicate gel. Thirty milliliter of n‐hexane was introduced into the chromatographic columns and eluted. The solvent composed of the mixture of 10% ether in n‐hexane has been introduced in all the column. The columns were covered with aluminum foil, and the concentrated solution containing the PAHs was introduced and rinsed three times with 2 ml of n‐hexane. The stopcock was opened, and hexane followed by 150 ml of solution was eluted quickly. The eluted solution was evaporated to 1–2 ml of solution. The residual solvent was then evaporated under nitrogen. At the end of this evaporation step, PAHs were dissolved in 1 ml of acetonitrile and injected into the HPLC‐FID. The estimated limit of detection (LOD) by HPLC ranged from 2.5 to 300 ppb.

### 2.4 Statistical analysis

Mean and standard deviation were determined on measurements. The data were also subjected to ANOVA with post‐test at 0.05 probability level. The Bonferroni test (compare all pair of columns) of the GraphPad InStat 3.1 software was used.

## 3 RESULTS

### 3.1 Effect of smoking processes on the proximate composition of fish

Tables 1 and 2 present the proximate composition of *C. gariepinus* and *C. carpio* after different smoking processes. Fishes are found to be rich in protein with values of 86.56 ± 3.09% and 88.65 ± 1.54%, respectively, for *C. gariepinus* and *C. carpio*. The lipid contents obtained were 10.46 ± 0.13% and 5.46 ± 0.11%, respectively, for *C. gariepinus* and *C. carpio*. There is a significant increase (*p* < 0.05) in the carbohydrate content of smoked fish. The lipid contents of fish smoked with *P. guajava* showed a significant increase (*p* < 0.05) of this parameter when compared to control. However, we found that fishes smoked with *P. guajava* without filter revealed higher lipid contents of 14.17 ± 0.15% and 14.96 ± 0.05%, respectively, for SNEGSF and SEGSF. The same observation has been made on *P. guajava* during the smoking of *C. carpio*.

**Table 1:** Proximate composition of *Clarias gariepinus* after smoking processes

	Protein content (% DM)	Lipid content (% DM)	Ash content (% DM)	Carbohydrate content (% DM)
*Control C. g*	86.56 ± 3.09^a^	10.46 ± 0.13^a^	1.92 ± 0.03^a^	0.94 ± 0.01^a^
SNE GSF	81.28 ± 1.54^b^	14.17 ± 0.15^b^	6.06 ± 0.30^b^	1.51 ± 0.14^b^
SE GSF	76.90 ± 1.54^c^	14.96 ± 0.05^b^	4.60 ± 0.10^c^	3.54 ± 0.04^c^
SNE MPF	76.91 ± 1.54^c^	13.02 ± 0.27^c^	4.53 ± 0.00^c^	5.54 ± 0.10^d^
SE MPF	74.72 ± 1.91^c^	13.80 ± 0.13^b^	4.60 ± 0.10^c^	6.88 ± 0.05^e^
SNE MPSF	76.90 ± 1.02^c^	14.91 ± 0.75^b^	6.02 ± 0.14^b^	1.90 ± 0.05^b^
SE MPSF	79.64 ± 0.77^b^	13.24 ± 0.33^c^	5.36 ± 0.09^d^	1.76 ± 0.14^b^
SNE MaSF	75.82 ± 3.09^c^	10.98 ± 1.72^d^	5.95 ± 0.23^b^	7.25 ± 0.14^e^
SE MaSF	72.08 ± 4.68^c^	12.55 ± 0.40^c^	5.76 ± 0.22^b^	9.61 ± 0.07^f^
SE GF	73.45 ± 0.77^c^	11.82 ± 0.06^d^	5.65 ± 0.11^b^	9.08 ± 0.06^f^
SNE GF	71.95 ± 0.64^d^	13.68 ± 0.21^b^	6.19 ± 0.06^b^	8.18 ± 0.04^f^
SE MaF	82.12 ± 0.00^b^	14.03 ± 0.12^b^	3.75 ± 0.17^e^	1.90 ± 0.05^b^
SNE MaF	79.81 ± 0.56^b^	14.18 ± 0.42^b^	4.89 ± 0.08^b^	1.12 ± 0.44^b^
SA	74.01 ± 3.41^c^	14.18 ± 0.42^b^	9.89 ± 0.02^f^	4.12 ± 0.04^c^
SNE FT	75.56 ± 1.54^c^	10.72 ± 0.18^d^	12.38 ± 0.81^g^	1.33 ± 0.27^b^
SE FT	75.46 ± 0.75^c^	12.25 ± 0.16^c^	11.21 ± 1.17^g^	6.29 ± 0.10^e^

Note

The value carrying different letters are significantly different (*p* < 0.05) from control and each other when compare all pairs of columns. Results presented are the means of two values followed by their standard deviation. *n* = 2.

Control *C. g*: control *C. gariepinus* (raw fish); DM: dry matter; SA: *C. gariepinus* not eviscerated traditional smoking oven purchased in the local market; SE FT: *C. gariepinus* eviscerated traditional smoking oven; SE GF: *C. gariepinus* eviscerated smoking with *Psidium guajava* with filter; SE GSF: *C. gariepinus* eviscerated smoking with *P. guajava* without filter; SE MaF: *C. gariepinus* eviscerated smoking with *Mangifera indica* with filter; SE MaPSF: *C. gariepinus* eviscerated smoking with *Rhizophora mangle* without filter; SE MaSF:* C. gariepinus* eviscerated smoking with *M. indica* without filter; SE MPF: *C. gariepinus* eviscerated smoking with *R. mangle* with filter; SNE FT: *C. gariepinus* not eviscerated traditional smoking oven; SNE GF: *C. gariepinus* not eviscerated smoking with *P. guajava* with filter; SNE GSF: *C. gariepinus* not eviscerated smoking with *P. guajava* without filter; SNE MaF: *C. gariepinus* not eviscerated smoking with *M. indica* with filter; SNE MaSF: *C. gariepinus* not eviscerated smoking with *M. indica* without filter; SNE MPF: *C. gariepinus* not eviscerated smoking with *R. mangle* with filter; SNE MPSF: *C. gariepinus* not eviscerated smoking with *R. mangle* without filter.

**Table 2:** Proximate composition of *Cyprinus carpio* after smoking processes

	Protein content (% DM)	Lipid content (% DM)	Ash content (% DM)	Carbohydrate content (% DM)
*Control C. c*	88.65 ± 1.54^a^	5.46 ± 0.11^a^	5.77 ± 0.05^a^	0.12 ± 0.14^a^
CNE GSF	85.46 ± 1.45^b^	7.99 ± 0.05^b^	4.52 ± 0.14^b^	2.03 ± 0.10^b^
CE GSF	85.31 ± 0.00^b^	9.81 ± 0.09^c^	4.21 ± 0.01^b^	0.67 ± 0.02^c^
CNE MPF	83.38 ± 3.09^c^	7.36 ± 0.62^b^	4.45 ± 0.22^b^	4.81 ± 0.09^d^
CE MPF	85.47 ± 1.58^b^	5.67 ± 0.07^d^	4.82 ± 0.07^b^	4.04 ± 0.11^d^
CE MPSF	86.66 ± 0.98^b^	7.57 ± 0.05^b^	4.54 ± 0.04^b^	1.23 ± 0.08^e^
CNE MPSF	85.56 ± 0.01^b^	7.09 ± 1.51^b^	4.44 ± 0.24^b^	2.91 ± 0.12^f^
CE MaSF	82.82 ± 0.77^c^	6.11 ± 0.09^e^	5.72 ± 0.36^a^	5.35 ± 0.12^d^
CNE MaSF	85.26 ± 0.01^b^	7.23 ± 0.96^b^	5.55 ± 0.09^a^	1.96 ± 0.02^b^
CNE GF	84.92 ± 0.82^b^	6.39 ± 0.11^e^	4.41 ± 0.03^b^	4.28 ± 0.09^d^
CE GF	82.83 ± 5.39^c^	6.86 ± 0.00^b^	4.34 ± 0.15^b^	5.97 ± 0.15^d^
CE MaF	81.90 ± 0.87^c^	6.65 ± 0.05^b^	4.80 ± 0.11^b^	6.65 ± 0.06^ed^
CNE MaF	84.92 ± 2.32^b^	3.93 ± 0.53^e^	4.95 ± 0.24^b^	6.2 ± 0.03^d^
CA	78.82 ± 1.41^d^	7.96 ± 0.05^bc^	4.60 ± 0.10^b^	8.54 ± 0.04^f^
CNE FT	86.20 ± 0.78^b^	6.07 ± 0.03^f^	5.97 ± 0.06^a^	1.24 ± 0.10^e^
CE FT	87.11 ± 0.77^a^	5.69 ± 0.02^d^	6.13 ± 0.24^a^	1.07 ± 0.04^e^

Note

The value carrying different letters are significantly different (*p* < 0.05) from control and each other when compare all pairs of columns. Results presented are the means of two values followed by their standard deviation. *n* = 2.

CA: *C. carpio* not eviscerated traditional smoking oven purchased in the local market; CE FT: *C. carpio* eviscerated traditional smoking oven; CE GF: *C. carpio* eviscerated smoking with *Psidium guajava* with filter; CE GSF:* C. carpio* eviscerated smoking with *P. guajava* without filter; CE MaF: *C. carpio* eviscerated smoking with *Mangifera indica* with filter; CE MaSF: *C. carpio* eviscerated smoking with *M. indica* without filter; CE MPF: *C. carpio* eviscerated smoking with *Rhizophora mangle* with filter; CE MPSF: *C. carpio* eviscerated smoking with *R. mangle* without filter; CNE FT:* C. carpio* not eviscerated traditional smoking oven; CNE GF: *C. carpio* not eviscerated smoking with *P. guajava* with filter; CNE GSF: *C. carpio* not eviscerated smoking with *P. guajava* without filter; CNE MaF: *C. carpio* not eviscerated smoking with *M. indica* with filter; CNE MaSF:* C. carpio* not eviscerated smoking with *M. indica* without filter; CNE MPF: *C. carpio* not eviscerated smoking with *R. mangle* with filter; CNE MPSF: *C. carpio* not eviscerated smoking with *R. mangle* without filter; Control *C. c*: control *C. carpio* (raw fish); DM: dry matter.

### 3.2 Effect of smoking processes on PAHs content

Tables 3 and 4 show the effect of different smoking processes on naphthalene, acenaphthene, fluoranthene, benzo (a) anthracene, chrysene, and benzo (a) pyrene content (ng/ml). We observed that the level of PAHs in the case of our study depends on the combination of treatments applied. *C. gariepinus* absorb more PAHs than *C. carpio*. We found that all the parameters such as the use of the filter, evisceration, and the type of smoking oven used impact on naphthalene and acenaphthene content, with a higher value of 1,451.54 ± 49.58 and 709.91 ± 8.12 ng/kg for traditional smoked *C. carpio*; and 1,841.12 ± 11.41 and 809.91 ± 1.10 ng/kg of traditional smoked *C. gariepinus*.

**Table 3:** Effect of the different smoking processes of *Cyprinus carpio* on the PAHs content

	Naphthalene (ng/ml)	Acenaphthene (ng/ml)	Fluoranthene (ng/ml)	Benzo (a) anthracene (ng/ml)	Chrysene (ng/ml)	Benzo (a) pyrene (ng/ml)
*Contrôle C. c*	3.14 ± 0.41^a^	8.11 ± 1.02^a^	nd	nd	nd	nd
CNE GSF	699.14 ± 12.10^b^	598.24 ± 12.01^b^	nd	nd	27.02 ± 10.10^a^	98.02 ± 2.10^a^
CE GSF	742.87 ± 14.10^c^	559.50 ± 10.10^b^	nd	nd	28.12 ± 07.10^a^	103.60 ± 07.10^a^
CNEMPF	124.11 ± 04.10^d^	124.41 ± 12.23^c^	nd	nd	nd	80.12 ± 1.10^b^
CE MPF	345.89 ± 7.10^e^	144.14 ± 10.10^d^	nd	nd	nd	87.12 ± 2.10^b^
CNE MPSF	487.25 ± 15.10^f^	241.87 ± 21.10^e^	nd	nd	nd	122.01 ± 1.12^c^
CE MPSF	784.12 ± 11.10^c^	300.14 ± 10.11^f^	nd	nd	nd	152.14 ± 7.10^d^
CNE MaSF	514.13 ± 12.01^g^	298.41 ± 12.11^f^	nd	nd	nd	144.12 ± 1.58^d^
CE MaSF	414.58 ± 22.25^h^	342.11 ± 8.21^g^	nd	nd	nd	135.11 ± 3.41^d^
CE GF	415.25 ± 10.14^h^	210.01 ± 1.12^e^	nd	nd	11.02 ± 1.23^b^	69.51 ± 1.02^e^
CNE GF	315.45 ± 15.78^e^	234.12 ± 8.21^e^	nd	nd	12.12 ± 2.12^b^	70.12 ± 1.99^e^
CE MaF	108.21 ± 05.10^d^	101.14 ± 12.10^c^	nd	nd	nd	50.44 ± 2.10^f^
CNE MaF	189.13 ± 15.40^i^	85.89 ± 1.52^f^	nd	nd	nd	65.21 ± 2.14^e^
CA	4,446.15 ± 88.41^j^	900.21 ± 14.12^g^	452.21 ± 2.10^a^	1,004.14 ± 08.14^a^	nd	598.42 ± 14.14^g^
CNE FT	1,451.54 ± 49.58^k^	709.91 ± 8.12^h^	314.12 ± 11.10^b^	641.14 ± 11.01^b^	119.31 ± 1.02^c^	621.12 ± 22.10^g^
CE FT	1,784.41 ± 14.12^l^	798.14 ± 10.10^i^	387.41 ± 11.12^b^	742.12 ± 2.12^c^	155.21 ± 2.01^d^	521.21 ± 1.09^h^

Note

The value carrying different letters are significantly different (*p* < 0.05) from control and each other when compare all pairs of columns. Results presented are the means of two values followed by their standard deviation. *n* = 2.

CA: *C. carpio* not eviscerated traditional smoking oven purchased in the local market; CE FT: *C. carpio* eviscerated traditional smoking oven; CE GF: *C. carpio* eviscerated smoking with *Psidium guajava* with filter; CE GSF: *C. carpio* eviscerated smoking with *P. guajava* without filter; CE MaF: *C. carpio* eviscerated smoking with *Mangifera indica* with filter; CE MaSF: *C. carpio* eviscerated smoking with *M. indica* without filter; CE MPF: *C. carpio* eviscerated smoking with *Rhizophora mangle* with filter; CE MPSF: *C. carpio* eviscerated smoking with *R. mangle* without filter; CNE FT:* C. carpio* not eviscerated traditional smoking oven; CNE GF: *C. carpio* not eviscerated smoking with *P. guajava* with filter; CNE GSF: *C. carpio* not eviscerated smoking with *P. guajava* without filter; CNE MaF: *C. carpio* not eviscerated smoking with *M. indica* with filter; CNE MaSF:* C. carpio* not eviscerated smoking with *M. indica* without filter; CNE MPF: *C. carpio* not eviscerated smoking with *R. mangle* with filter; CNE MPSF: *C. carpio* not eviscerated smoking with *R. mangle* without filter; Control *C. c*: control *C. carpio* (raw fish); DM: dry matter.

**Table 4:** Effect of different smoking processes of *Clarias gariepinus* on the PAHs content

	Naphthalene (ng/ml)	Acenaphthene (ng/ml)	Fluoranthene (ng/ml)	Benzo (a) anthracene (ng/ml)	Chrysene (ng/ml)	Benzo (a) pyrene (ng/ml)
*Contrôle C. g*	4.87 ± 0.77^a^	11.10 ± 1.24^a^	nd	nd	nd	nd
SNE GSF	798.45 ± 47.14^b^	669.21 ± 0.78^b^	nd	nd	27.77 ± 1.04^a^	107.40 ± 1.10^a^
SE GSF	800.41 ± 11.21^b^	669.32 ± 1.41^b^	nd	nd	30.12 ± 2.05^a^	107.40 ± 2.10^a^
SNE MPF	135.98 ± 0.41^C^	124.10 ± 2.45^c^	nd	nd	nd	90.14 ± 2.12^b^
SE MPF	138.54 ± 11.41^c^	152.14 ± 8.41^d^	nd	nd	nd	98.44 ± 3.12^c^
SE MPSF	452.21 ± 21.10^d^	254.14 ± 10.12^e^	nd	nd	nd	122.22 ± 14.01^d^
SNE MPSF	641.01 ± 45.11^f^	240.14 ± 10.12^e^	nd	nd	nd	251.20 ± 10.02^e^
SE MaSF	451.21 ± 11.45^d^	412.11 ± 4.41^f^	nd	nd	nd	122.25 ± 7.12^d^
SNE MaSF	435.12 ± 30.01^d^	354.11 ± 5.21^g^	nd	nd	nd	156.21 ± 2.14^f^
SNE GF	258.41 ± 7.41^g^	331.12 ± 7.10^h^	nd	nd	10.10 ± 0.89^b^	65.10 ± 1.05^g^
SE GF	344.11 ± 10.41^h^	241.01 ± 12.01^e^	nd	nd	9.11 ± 1.02^b^	74.10 ± 0.12^h^
SE MaF	125.12 ± 11.78^c^	118.29 ± 1.10^c^	nd	nd	nd	79.12 ± 1.20^i^
SNE MaF	142.58 ± 14.10^c^	102.78 ± 4.12^c^	nd	nd	nd	64.12 ± 0.78^g^
SA	375.12 ± 11.14^h^	880.14 ± 25.14^i^	423.11 ± 11.10^a^	1,102.52 ± 54.01^a^	nd	621.00 ± 22.10^j^
SNE FT	1,841.10 ± 11.41^i^	809.91 ± 1.10^i^	541.12 ± 10.15^b^	974.41 ± 12.14^b^	nd	587.21 ± 10.10^k^
SE FT	1,998.12 ± 12.01^j^	909.93 ± 09.74^j^	521.22 ± 02.11^c^	977.12 ± 25.10^b^	nd	669.60 ± 11.02^l^

Note

The value carrying different letters are significantly different (*p* < 0.05) from control and each other when compare all pairs of columns. Results presented are the means of two values followed by their standard deviation. *n* = 2.

Control *C. g*: control *C. gariepinus* (raw fish); DM: dry matter; SA: *C. gariepinus* not eviscerated traditional smoking oven purchased in the local market; SE FT: *C. gariepinus* eviscerated traditional smoking oven; SE GF: *C. gariepinus* eviscerated smoking with *Psidium guajava* with filter; SE GSF: *C. gariepinus* eviscerated smoking with *P. guajava* without filter; SE MaF: *C. gariepinus* eviscerated smoking with *Mangifera indica* with filter; SE MaPSF: *C. gariepinus* eviscerated smoking with *Rhizophora mangle* without filter; SE MaSF:* C. gariepinus* eviscerated smoking with *M. indica* without filter; SE MPF: *C. gariepinus* eviscerated smoking with *R. mangle* with filter; SNE FT: *C. gariepinus* not eviscerated traditional smoking oven; SNE GF: *C. gariepinus* not eviscerated smoking with *P. guajava* with filter; SNE GSF: *C. gariepinus* not eviscerated smoking with *P. guajava* without filter; SNE MaF: *C. gariepinus* not eviscerated smoking with *M. indica* with filter; SNE MaSF: *C. gariepinus* not eviscerated smoking with *M. indica* without filter; SNE MPF: *C. gariepinus* not eviscerated smoking with *R. mangle* with filter; SNE MPSF: *C. gariepinus* not eviscerated smoking with *R. mangle* without filter.

A significant increase (*p* < 0.05) in PAHs levels at the end of smoking processes has been recorded. The different smoking treatments, when compared to each other, revealed a significant difference (*p* < 0.05) between fish smoked with traditional smoking oven and fish smoked with improved smoking oven. The total PAHs content was higher in fish smoked with traditional smoking oven. There was also a significant decrease (*p* < 0.05) in PAHs levels after utilization of filter. Fluoranthene and Benzo(a)anthracene were not identified in fish smoked fish with ameliorated smoking oven. However, high levels of these compounds were still observed in fish bought on the market and into those smoked on a traditional smoking oven.

## 4 DISCUSSION

### 4.1 Effect of different smoking processes on the proximate chemical composition

Fish can be classified according to their lipid content as follows: lean fish (lipid content is <5%), semifat fish (lipid content range between 5% and 10%), and fat fish (lipid content is higher than 10%; Suriah, Huah, & Duad 1995). Based on this classification, *C. gariepinus* can be classified as a fat fish and *C. carpio* as a semifat fish. After smoking processes, a significant increase (*p* < 0.05) in the lipid content was observed. However, the protein contents show a significant decrease (*p* < 0.05). *C. carpio* smoked with *P. guajava* using a filter shows a slight increase in the lipid content (*p* < 0.05). In fact, levels of 6.36 ± 0.11 and 5.86 ± 0.00 were recorded after filter smoking, for CNEGF and CEGF, respectively. The increases observed are due to the water losses observed during the smoking process which allows concentration of these nutrients. Indeed, Arason, Nguyen, Thorarinsdottir, and Thorkelsson (2014) observed that different treatment methods can affect the nutritional composition of fish. These effects can be negative in the long term if the consumption of the latter is excessive. In addition, Chukwu and Shaba (2009) point out that heating, smoking, freezing, and exposure to high salt concentrations lead to chemical and physical changes, which increase the digestibility of proteins. These changes also reduce thermolabile compounds and polyunsaturated fatty acids. The increase in lipids content observed with *C. carpio* and *C. gariepinus* smoked can be explained by the reduction in moisture content. This is consistent with previous findings, which reported an inverse correlation between fat and water levels, common for many fish species (Ljubojevic et al., 2016; Zmijewski, Kujawa, Jankowska, Kwiatkowska, & Mamcarz, 2006).

### 4.2 Effect of smoking processes on the PAHs content of fish

Traces of naphthalene and acenaphthene found in raw fish of 3.14 ± 0.41 and 8.11 ± 1.02 ng/kg in *C. gariepinus*, and of 4.78 ± 0.77 and 11.10 ± 1.24 in *C. carpio*, respectively, come from the growing environment. The trace of naphthalene and acenaphthene identified in raw fish shows that PAHs obtained in this study come exclusively from woods pyrolysis involved in the smoking processes. Indeed, Stolyhwo and Sikorski (2005) revealed that fish and marine invertebrates have low levels of PAHs or undetectable amounts absorbed from the environment. PAH content differs according to the fish species. These significant differences (*p* < 0.05) can be explained by differences in lipid composition of fishes, related to their lipophilic nature as revealed by Faham (2013). Indeed, Nakamura et al. (2008) in their studies on the pyrolysis of lignin revealed that skin is an important parameter during smoking that limits the absorption of PAHs. In the case of this study, the use of the metallic filter leads to the reduction in PAHs content. In fact, the rack underneath reduces the temperature of smoke and, thus, reduces the rate of convection of aromatic compounds that migrate into fish. Low molecular weight PAHs with 2–3 cycles are in gaseous form while high molecular weight PAHs with 5–6 cycles are adsorbed as fine particles because of their hydrophobicity and low volatility. The intermediate molecular weight PAHs of 4 cycles will be distributed between the two phases (Ineris & Quiot, 2005). PAHs are relatively nonvolatile and not very soluble in water. They cannot evaporate easily from the materials that contain it. Thus, during our different smoking processes, wood carbonization observed from 400°C as revealed by Visciano et al. (2008) lead to the formation of PAHs in smoke. However, parts of PAHs containing more than four aromatic rings are deposited on the metallic filter. The level of PAHs retained by the metallic filter would therefore increase proportionally with the duration of the smoking processes. This retention by the filter leads to a decrease in PAH content in smoked fish and explains the significant decrease (*p* < 0.05) of PAH compounds in fish smoked using filters.

The concentration of PAHs depends on the density of smoke, the availability of air, the process duration, and the surface of the product to be smoked and especially on the smoking temperature. The decrease in temperature may also lead to the reduction in the smoke density. In fact, according to Pöhlmann, Hitzel, Schwägele, Speer, and Jira (2012), the density of smoke impact on the polycyclic aromatic hydrocarbon content of the finished product. Indeed, molecules of high molecular weight require high temperatures to remain in vapor phase, which facilitates their absorption by the product as reported by Girard (1988). The lyophilic character of PAHs is responsible for their accumulation in lipids. The higher lipid content in *C. gariepinus* has facilitated this transfer and explains his high levels. The PAH composition varies according to the type of wood used. The naphthalene content in the case of this study depends on the wood species used, and difference in composition would then affect his pyrolysis.

According to Mohd, Kawamoto, and Shiro (2010), the chemical structure of lignin and hemicellulose differs according to the type of wood used particularly softwood and hardwood. Evisceration significantly increased (*p* < 0.05) the PAHs content by increasing the contact surfaces between smoke and pretreated fish. Results obtained in this study show that smoking using metallic filter of mesh 100 µm lead to smoked product of PAHs content under the European limit.

## 5 CONCLUSION

This work was designed to study the effect of different smoking processes on the nutritional and PAHs composition of smoked *C. gariepinus* and *C. carpio*. The smoking process was conducted using vegetal material such as *P. guajava*, *R. mangle*, and *M. indica*, a traditional and ameliorated smoking oven and two fish species. After smoking processes, lipid content increases the PAHs content due to his lipophilic property. PAHs migrations in finish products were reduced when processes were carried out using a metallic filter. The evisceration and the lipid content of fish increase the PAHs content in the finished product. Then, the quality of smoked product is improved with the metallic filter of 100 µm during smoking processes.

## CONFLICT OF INTEREST

The authors declare that they do not have any conflict of interest.

## AUTHOR CONTRIBUTIONS

C.T. Tiwo, F. Tchoumbougnang, and E. Nganou have designed the research work. C.T. Tiwo performed smoking and drafted the manuscript. C.T. Tiwo, B. B. Nayak, and K. Pankaj carried out the research work by determining the proximate chemical composition of smoked fish and the PAH content in smoked fish. All authors read and approved the final manuscript.

## ETHICAL APPROVAL

This study does not involve any human or animal testing.
